# Mental Fatigue Affects Visual Selective Attention

**DOI:** 10.1371/journal.pone.0048073

**Published:** 2012-10-31

**Authors:** Léon G. Faber, Natasha M. Maurits, Monicque M. Lorist

**Affiliations:** 1 Department of Experimental Psychology, University of Groningen, Groningen, The Netherlands; 2 BCN Neuroimaging Center, University of Groningen, Groningen, The Netherlands; 3 Department of Neurology, University Medical Center Groningen, University of Groningen, Groningen, The Netherlands; Radboud University Nijmegen, The Netherlands

## Abstract

Mental fatigue is a form of fatigue, induced by continuous task performance. Mentally fatigued people often report having a hard time keeping their attention focussed and being easily distracted. In this study, we examined the relation between mental fatigue, as induced by time on task, and attention-related changes in event-related potentials (ERPs). EEG, reaction times and response accuracies were obtained from 17 healthy volunteers during two hours of task performance on an adapted Eriksen flanker task. In this task, the size of targets and flankers was manipulated to discern neuronal processes that are related to processing of relevant information from processes related to the processing of irrelevant information. The ERP data showed that effects induced by target size manipulation were not affected by time on task, while an initial effect of flanker size manipulation decreased gradually with increasing time on task. We conclude that attention was affected by mental fatigue, in the form of a decrease in the ability to suppress irrelevant information. In behavioural results, this was reflected by a tendency of participants to increasingly base their response decision on irrelevant information, resulting in decreased response accuracies.

## Introduction

When people are working on a cognitively demanding task for a prolonged period of time, they will often experience mental or cognitive fatigue, reflected in deteriorated task performance and reduced motivation to continue to work on the task at hand [1]–[3]. Moreover, an increase in the amount and severity of errors being made can generally be observed. An important observation is that mentally fatigued people often report having a hard time keeping their attention focused and that they are easily distracted (e.g., [4]), indicating that mental fatigue could have effects on selective attention.

Several studies have examined the relation between mental fatigue and selective attention. For example, van der Linden and Eling [5] looked at the effects of mental fatigue on local versus global processing in a local-global task. They found that during conditions of mental fatigue, global shape processing suffers less from mental fatigue than local shape processing, which relies more strongly on top-down – attention-guided – processing. In addition, Boksem et *al*., [6] showed that several attention-related ERP components were affected by mental fatigue. In their experiment, participants had to detect a target, if it was presented on a relevant diagonal. An interesting effect they found was that the N2b showed an increased negativity with time on task and that an initial N2b difference they observed between stimulus presentations on the relevant and irrelevant diagonal decreased with time on task.

These studies deliver clear indications that selective attention is indeed influenced by mental fatigue. The present study further investigates the influence of mental fatigue on the selection of relevant versus irrelevant information by examining both changes in the processing of relevant stimulus information as well as in the processing of irrelevant stimulus information. To accomplish this, we used a method that allows separating the effects of mental fatigue on both types of information, while presenting them simultaneously.

We applied the principle that selective attention can be considered a top-down controlled neuronal gain mechanism, boosting relevant signals, while at the same time attenuating irrelevant signals [7]–[12]. This top-down mechanism feeds back into visual processing areas as far back as area V1, as has been shown by single cell recordings in macaques [13]–[18], fMRI research [19]–[23] and EEG experiments in humans [24], [25]. In lower visual processing areas, attention has a fill-in effect, meaning that after the initial response to a stimulus presentation, neurons with a receptive field within the area occupied by an attended stimulus, after a relatively long delay, will become more strongly activated due to attention-related top-down modulations, while activity levels of neurons which have a receptive field within the area occupied by unattended stimuli become attenuated [26]–[29]. We combined this with the principle that in lower visual processing areas, neurons have smaller receptive fields than in higher visual processing areas [30]. Therefore, in a lower visual processing area, the size of a stimulus is more strongly reflected in the number of neurons that become excited by the stimulus than in higher visual processing areas of the brain.

Based on these properties of the visual processing system, we can predict that besides an early direct modulation of activity in the EEG, caused by the size of the stimulus (i.e. larger stimuli activate more neurons and more pronounced EEG activity), the size of a stimulus will also elicit a late, indirect effect. The initial direct effect is elicited by the processing of feed-forward activations from the lateral geniculate nucleus of the thalamus (LGN), caused by stimulus onset. The late indirect effect is caused by the number of neurons being disinhibited and inhibited by attention in visual processing areas, as shown by Scholte et *al*., [31] in an ERP study of figure-ground segregation in which figure size was manipulated. Scholte et *al*. found an early effect of surface segmentation after 112 ms on temporal electrodes, reflecting high-level visual processing. After 172 ms effects of surface segmentation were found on occipital electrodes, which reflect activation in those areas that are lowest in the visual processing hierarchy. Based on these sequential ERP effects they concluded that surface segregation effects concerns a feedback process; first taking place in higher order brain areas and then propagate backwards along the visual processing stream.

In the current experiment we used an adapted version of the Eriksen flanker task [32], in which we varied the size of both the relevant targets and the irrelevant flankers. An important difference between the current experiment and figure-ground segregation experiments is that in the figure-ground segregation paradigm, the stimulus as a whole does not vary in size. Therefore, in a figure-ground segregation paradigm, there is little or no direct effect of size on early ERP components, caused by differences in unmodulated feedforward activations. Only the indirect effect caused by top-down neuronal disinhibition will show in the ERP. Instead, in the current experiment we compare size manipulations of both the target (relevant information) and the flankers (irrelevant information), though in separate trials.

Manipulating stimulus sizes globally, as is done in the present study, will cause direct effects, due to differences in the initial feed-forward response to (retinal) signals from the LGN. These direct effects will be reflected in early ERP components, such as the P1, known to reflect qualitative properties of stimuli [33]. Our manipulation also allows us to distinguish between the enhancing effect that selective attention has on relevant signals and the attenuating effect it has on irrelevant signals. These effects should be reflected later in the ERP than the direct effects, because they are dependent on feedback from higher brain areas [34].

We expect that changes in selective attention due to mental fatigue, will be reflected in gradual changes in the indirect effect. Thus we expect the difference in the late ERP elicited by the target size manipulation to show a different pattern through time than the difference elicited by the flanker size manipulation. Note that behavioural results cannot be interpreted in the same manner as ERPs. Overt responses are the end product of a series of processes, leading to the accumulation of evidence [35], resulting in a decision which response is executed. Although behaviour is, among others, depend on selective attention mechanisms; it does not exclusively reflect these processes. ERPs provide a more direct reflection of the specific processes executed in the brain than behaviour. Because we are specifically interested in differential effects of target processing versus flanker processing in the present experiment, ERPs provide essential information in addition to our behaviour measures.

## Methods

### Ethics Statement

The study was approved by the Medical Ethical Committee of the University Medical Center Groningen (METc 2008/323) and the experiment was undertaken in compliance with national legislation and the Declaration of Helsinki. Participants gave written informed consent prior to the measurements.

### Participants

Seventeen healthy volunteers (10 females, mean age: 20.4, SD: 2.9) participated in the study. All participants had normal sleeping patterns, had normal or corrected to normal vision and were right handed. Volunteers were excluded from participation if they had neurological or psychiatric complaints, or if they used medication or drugs that might affect task performance.

### Procedure

Participants came to the laboratory on two consecutive days. On the first day each participant received a training session consisting of six practice blocks of five minutes of the experimental task. After each practice block, the participants received feedback about their performance level. If they performed within the range of 85–95% correct responses, the feedback would be that their performance was sufficient. If the percentage of correct trials was lower than 85%, they were asked to increase their accuracy and if the percentage of correct trials was above 95% they were asked to react faster.

On the second day the experimental session took place. Participants were instructed to abstain from coffee and other caffeine containing substances for eight hours preceding the experiment and from alcohol 24 hours preceding the experiment. After arrival at the laboratory at 9∶30 AM, participants handed over their watches and cell phones. They had no knowledge of the length of the experiment or the experimental blocks, other than that it would not take longer than 3.5 h in total. After the application of the electrodes, participants were seated 75 cm from the screen, inside a sound attenuated dimly lit (∼15 lux, measured in front of the display) room.

### Experimental Task and Stimuli

The experiment consisted of six blocks of 20 minutes, during which the participant had to perform a reaction time task, which was a variation of the Eriksen flanker task [32]. A target letter was placed between distracter letters or flankers, two on either side. The letters used in the flanker task were the letters ‘H’ and ‘O’, presented in white on a black background for 70 ms. Stimuli could be congruent, meaning that all stimulus letters had the same identity (i.e. HHHHH) or incongruent, meaning that flanker letters had another identity than the target (i.e. OOHOO). Stimuli were presented on a CRT computer monitor running at 1024×768 px @ 85 Hz. The inter-stimulus intervals were randomly chosen between 2000 and 2500 ms.

We are interested in signals from the lower cortical visual processing areas; in these processing areas neuronal receptive field sizes are smaller compared to higher level visual processing areas, and therefore stimulus size is expected to be most strongly reflected in neuronal activation in these areas. However, some early visual processing areas lie in the posterior part of the brain, surrounding the calcarine and medial fissures. Stimuli from each quadrant of the retinal image are projected on opposing banks of these fissures. The neurons in the cortical sheet on the opposing banks are oriented in opposing directions, causing electrical potentials, which can largely cancel out each other, when they occur simultaneously [36], [37]. Therefore in the present study each stimulus will be presented inside a single quadrant of the visual field [24], [25]. To make sure that participants fixate on the fixation cross and not on the location where the stimulus appears, two locations for stimulus presentation were chosen. Evidence exists that the horizontal midline, like the upper hemifield, is projected onto the lower bank of the calcarine fissure [36], [37]. To avoid having parts of the lower hemifield stimuli projected onto the lower bank, only upper hemifield stimuli were used.

**Figure 1:**
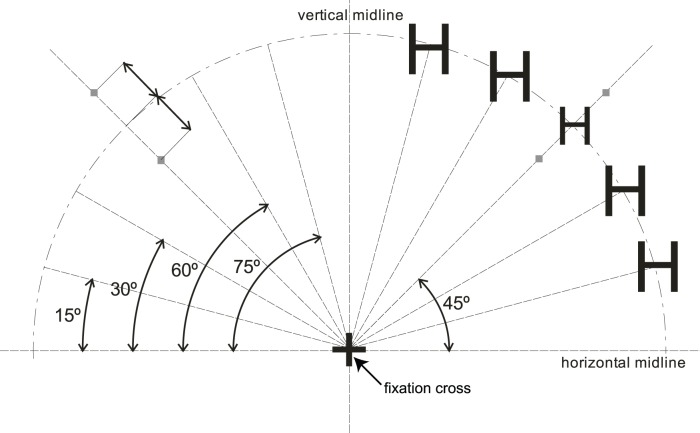
Stimulus screen layout with an example of a right side stimulus. All letters of the stimulus are placed equidistant from the fixation cross, equally spaced from each other.

The letters used in the flanker task were presented in a bold Arial font and all letters in each stimulus presentation were arranged in a quarter circular arrangement in the left upper or right upper quarter of the screen, equidistant at 3.4° visual angle from the fixation cross and equally spaced from each other at angles of 15°, 30°, 45°, 60° and 75° from the horizontal midline (see Figure 1). The fixation cross was constantly visible in the centre of the screen during the experimental blocks and participants were instructed to keep their eyes focussed on it. On the inside and outside of the two possible target locations, small grey dots were placed, in order to make it easier for participants to identify the location of the target.

Stimulus letters were presented in three possible sizes, small, medium or large, of 0.36°, 0.52° or 0.72° visual angle, respectively. Trials were divided into target trials and flanker trials. In the target trials, used to examine effects of selective attention on the processing of relevant information, the size of the target stimulus was manipulated and thus presented as small or large sized and all flankers were presented medium sized. In flanker trials, used to examine effects of processing of irrelevant information, the size of the flankers was manipulated and they were either all small or all large sized, while the target was presented medium sized in these trials. Note that in all trials there were size differences between flankers and targets, in order to avoid differences in the pop-up effect between trials. Each stimulus type was chosen randomly with equal probability.

Participants had to report the identity of the target letter, by pressing a corresponding button; e.g. left button press with the left index finger for an ‘H’ and right button press with the right index finger for an ‘O’. The assignment of buttons to letters was pseudo randomised per participant. Subjects were told to do this as fast as possible while maintaining high accuracy similar to how they were trained the day before. The participants’ reactions were recorded by means of a response box, connected to the serial port of the stimulus computer.

Before each 20 minute block, participants had to answer the question, “How much resistance do you feel towards performing on the upcoming task?” on a scale from 0 (‘None at all’) to 9 (‘Extremely much’) (adapted from [38]) via a keyboard. They had at most five seconds to give a rating (indicated by an audible signal). Immediately after the rating the experimental task would continue, giving an effective break of approximately five seconds, making the task nearly continuous.

### EEG Recordings

EEG data were recorded using a 62-channel electrode cap from Electro Cap International with Sn electrodes placed according to the 10–10 system. Signals were recorded using a TMSI Refa common reference amplifier with 20-bit resolution. Signals from separate Sn electrodes placed on the left and right mastoids were recorded for off-line referencing. The electro-oculogram was recorded bipolarly, using Sn electrodes placed on the outer canthi of both eyes and above and below the left eye. Participants were grounded by a Sn electrode placed on the sternum. All impedances were kept below 5 KΩ. Signals were sampled at 500 Hz using Brain Vision Recorder (Brain Products GmbH) software.

### Data Analysis

Statistical analyses of subjective ratings, mean reaction times, mean proportions accurate responses, and mean ERP deflections were performed using a repeated measures ANOVA with the factors time-on-task (intervals of 20 min), congruency (congruent/incongruent), relevance (target versus flanker manipulation) and size (large/small). When the sphericity assumption was not met, a Huyn Feldt correction was applied when epsilon was larger than 0.75 and a Greenhouse Geisser correction was applied otherwise. Polynomial within subjects contrasts were computed to analyse trends in time-on-task effects. When (post hoc) multiple comparisons were performed, Bonferroni corrections were applied. A significance level of α = 0.05 was assumed.

In this experiment, the size manipulation is used to investigate effects of stimulus processing on low-level visual processing areas. The factor relevance reflects no qualitative stimulus aspects, but refers to which part of the total stimulus – relevant or irrelevant – the size manipulation refers to. It is therefore not very meaningful to study relevance effects, without considering its interaction with size manipulation. Therefore, main effects of relevance or any interactions that contain relevance as a factor in which size is not included as factor as well are not included in the statistical model. These interactions will also not be mentioned in tables.

### Behavioural Data

Average accuracy and reaction times of correct responses were computed for each participant in each condition in each block. Responses were considered correct if the correct button response was hit between 150 and 1400 ms after stimulus onset. All other responses were considered incorrect.

### Event-related Potentials

ERP analyses were performed using Brain Vision Analyzer (Brain Products GmbH). Raw EEG was referenced to averaged mastoids and then band pass filtered, using a high-pass Butterworth filter at 0.16 Hz, 48 dB/Oct and a low pass filter at 40 Hz, 48 dB/Oct. Continuous data were segmented into epochs from 200 ms pre-stimulus to 1000 ms post-stimulus. Epochs in which participants made premature responses (<150 ms), late responses (>1400 ms) or incorrect button presses were discarded. Next, artefact rejection (maximal allowed voltage step/sampling point: 50 µV, maximal allowed absolute difference between any two values in the segment: 300 µV, maximal allowed amplitude: +/−200 µV, maximal amplitude EOG channels: +/−1200 µV) and ocular correction [39] were applied. Finally, epochs were averaged separately for each experimental condition and a baseline correction was applied. The factors time-on-task (20 minute blocks), congruency, relevance (target-versus-flanker trial manipulation) and size were analysed. Because behavioural results revealed a remaining learning effect during the first 20-minute block, the first block was excluded from the ERP analysis.

Statistical analyses of the ERPs were performed on mean amplitudes in fixed intervals. Based on visual inspection of grand average ERPs, mean amplitudes for different components were calculated in the following intervals at the following electrode positions: for the contralateral P1 between 90 and 130 ms (O1 and O2), for the ipsilateral P1 between 120 and 160 ms (O1 and O2), for the contralateral N1 between 140 and 190 ms (P7 and P8), for the P2 between 210 and 250 ms (PO3 and PO4), for the N2b between 260 and 300 ms (Cz) and for the P3b between 400 and 480 ms (Pz) for each subject and each condition. Lateralised effects were analysed by averaging the ERPs from homologue electrode pairs (e.g. P7, P8), where ipsilateral and contralateral refers to scalp positions at electrodes ipsilateral and contralateral, respectively, to the side where the stimulus was presented. Visual inspection indicated that the ascending flank of the P3b in the 300–400 ms interval was modulated by task manipulations, therefore an additional statistical analysis was performed on the mean amplitudes in this interval at CPz, where the effect was most pronounced.

## Results

### Subjective Fatigue

Subjective fatigue as measured by the resistance questionnaire showed a significant increase from 1.7 (SD = 2.3) before the first experimental block to 7.3 (SD = 2.2) before the last experimental block (F(5, 85) = 30.00, P<.001).

### Behavioural Measures

**Figure 2:**
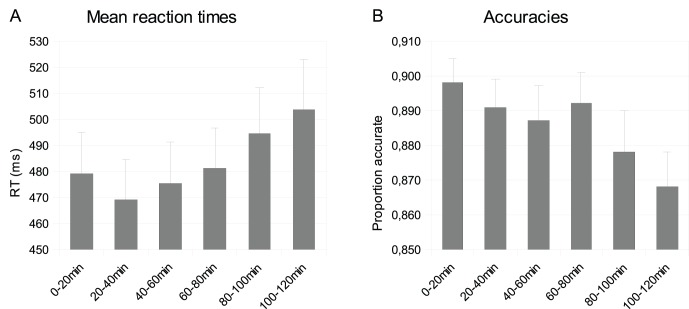
Mean reaction times (A) and accuracies (B) for each 20 min block. Error bars represent standard errors.

Reaction times (RTs) increased with time-on-task (F(5, 80) = 5.780, p = .007), as is shown in Figure 2A, though besides a linear increase (F(1, 16) = 6.51, p = .021) a U-shaped quadratic trend can also be observed (F(1, 16) = 6.349, p = .023), due to a decrease in reaction times during the first 20 minutes. Because of this, the first 20-min block was excluded from the ERP analyses. The mean proportion of accurate responses decreased with time-on-task from.891 to.864 (F(5, 80) = 3.91, p = .025) in a linear fashion (F(1, 16) = 5.51, p = .032; Figure 2B).

Participants reacted faster and more accurately to congruent stimuli (RT = 464 ms, SD = 60.6; accuracy = .937, SD = .029) than to incongruent stimuli (RT = 504, SD = 71.9; accuracy = .83, SD = .062; F(1, 16) = 56.84, p<.001 and F(1, 16) = 37.64, p<.001 for RT and accuracy, respectively). The increase in reaction times and decrease in accuracy with time on task on reaction times were similar for congruent and incongruent stimuli (time on task × congruency: F(5, 80) = 1.416, n.s.). The difference in accuracy between congruent and incongruent trials increased over time (time on task × congruency: F(5, 80) = 4.55, p = .001). Additional analyses showed that time on task effects negatively affected accuracy on incongruent trials (F(5, 80) = 4.57, p = .011), but not on congruent trials (F(5, 80) = 2.52, n.s.).

**Figure 3:**
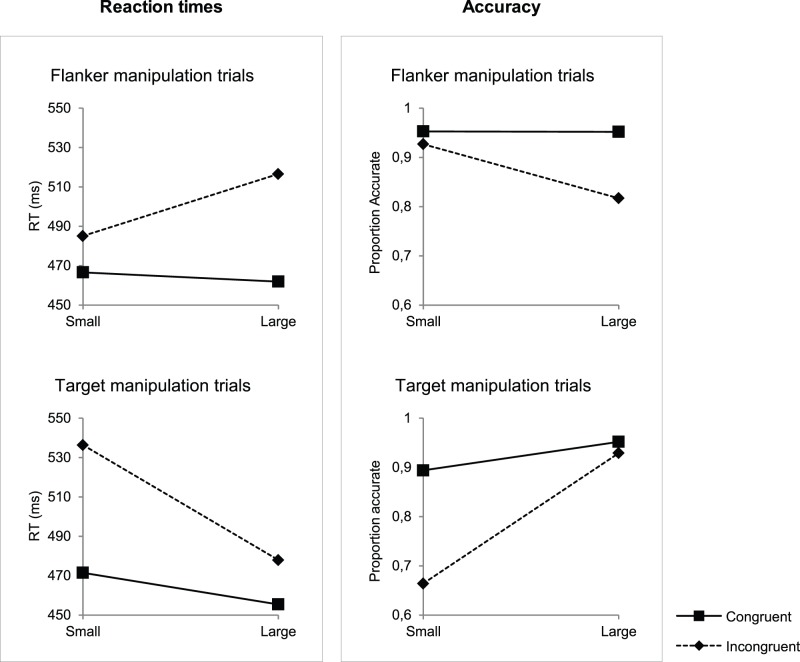
Interaction plot of size × relevance × congruency. Three way interactions between size, relevance (target versus flanker manipulation) and congruency, on reaction times and response accuracies are illustrated. The solid lines with the squares represent the congruent condition and the dotted lines with the diamonds represent the incongruent condition.

In general, we observed that if targets were relatively large compared to the flanker letters, responses were faster (target manipulation trials: F(1, 16) = 383.26, p<.001; flanker manipulation trials: F(1, 16) = 52.04, p<.001) and more accurate (target manipulation trials: F(1, 16) = 102.11, p<.001; flanker manipulation trials: F(1, 16) = 37.92, p<.001) compared to the conditions in which targets were smaller than the flankers (see Figure 3). More specific, the effect of the size manipulation is opposite for trials in which the size of the flankers was manipulated compared to trials in which the target size was manipulated for both reaction times (F(1, 16) = 227.85, p<.001) and accuracy (F(1, 16) = 89.18, p<.001). In the flanker size manipulation condition people reacted faster (F(1, 16) = 52.04, p<.001) and more accurately (F(1, 16) = 37.92, p<.001) in trials with small flankers than in trails with large flankers. In the target size manipulation condition people reacted faster (F(1, 16) = 383.26, p<.001) and more accurately (F(1, 16) = 102.11, p<.001) to large targets than to small targets. As shown in Figure 3, differences in accuracy and RTs between congruent and incongruent trials were found to be more pronounced if targets were relatively small compared to the flankers (F(1, 16) = 49.50, p<.001 and (F(1, 16) = 34.30, p<.001, for reaction times and accuracy, respectively). Post-hoc analyses showed that when targets were relatively large, accuracy and RT differences between congruent and incongruent stimuli did remain significant (small flankers RT: F(1, 16) = 35.52, p<.001; small flankers accuracy: F(1, 16) = 58.85, p<.001; large targets RT: F(1, 16) = 43.12; p<.001; large targets accuracy: F(1, 16) = 6.53; p = .021). Alternatively, this effect can be interpreted as a reduced size effect in the congruent condition compared to the incongruent condition; size differences in the congruent condition were significant (RT: F(1, 16) = 43.77, p<.001; accuracy: F(1,16) = 28.98, p<.001). On congruent flanker manipulation trials an effect of size manipulation was absent (RT: F(1, 16) = 5.46, n.s.; accuracy: F(1, 16) = .02, n.s.).

**Figure 4:**
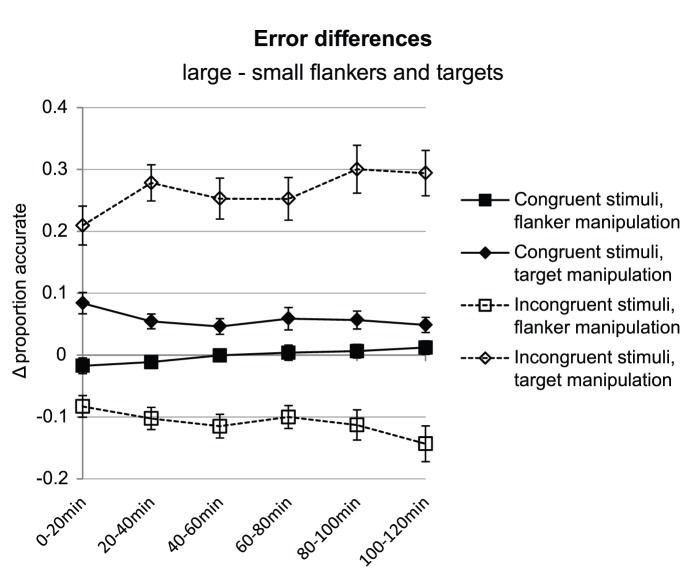
for incongruent stimuli (dotted lines) and decrease or remain constant for congruent stimuli (solid lines). Note that directions of the target size manipulation condition (positive) are opposite to those of the flanker size manipulation condition (negative or neutral). From the perspective of relative target to flanker size, they would be in the same direction. Error bars represent standard errors.

For accuracies, the difference in the size effect between congruent and incongruent trial increased with time on task (time on task × congruency × relevance × size: F(5, 80) = 6.69, p<.001). A more simple way to describe this four-way interaction is as a three-way interaction between time on task, congruency, and relative target size (F(5, 80) = 6.69, p<.001), since the two relevance conditions of this four-way interaction produce similar effects at opposite sides of the horizontal axis. As is shown in Figure 4, the difference in accuracy between relatively large and small targets increased for incongruent stimuli on both the target- and the flanker size manipulation trials (F(5, 80) = 4.17, p = .002; F(5, 80) = 2.82, p = .021, respectively), while no significant size-related changes were observed for congruent stimuli (target manipulation trials: F(5,80) = 1.39, n.s.; flanker manipulation trials: F(5, 80) = 1.33, n.s.).

An overview of the behavioural results can be found in Table 1.

**Table 1:** Main and interaction effects on reaction times and accuracies.

		Reaction times		Accuracy	
Factors	df	F	p	F	p
TOT	5, 80	5.780	.007	3.905	.025
Cong	1, 16	56.836	<.001	37.637	<.001
Size	1, 16	195.724	<.001	77.976	<.001
TOT×Cong	5, 80	1,416		4.552	.001
TOT×Size	5, 80	1.519		.914	
Cong×Size	1, 16	1.739		16.146	<.001
TOT×Cong×Size	5, 80	0.231		1.145	
Rel×Size	1, 16	227.845	<.001	89.176	<.001
TOT×Rel×Size	5, 80	.490		1.791	
Cong×Rel×Size	1, 16	49.5	<.001	34.300	<.001
TOT×Cong×Rel×Size	5, 80	.672		6.692	<.001

TOT  =  time on task, Cong  =  congruency, Rel  =  relevance. Only significant p-values are listed.

### Event-related Potentials

#### P1

**Figure 5:**
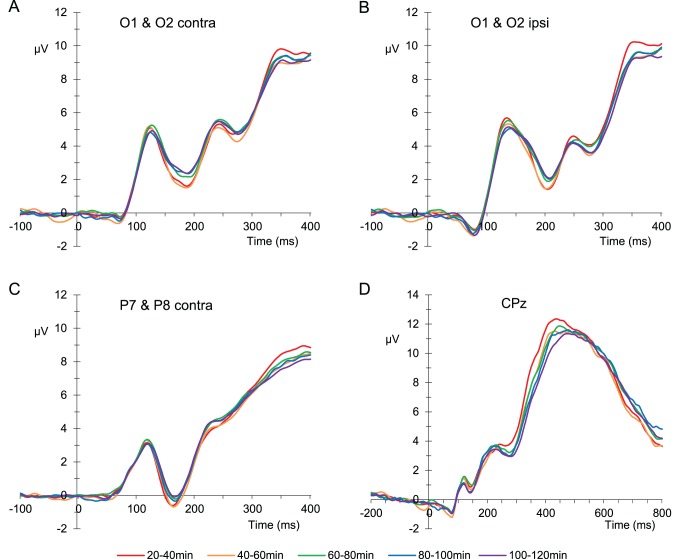
ERPs in time on task intervals. A) ERPs of experimental blocks, averaged over electrode positions O1 and O2 contralateral to stimulus presentations. B) ERPs of experimental blocks, averaged over electrode positions O1 and O2 ipsilateral to stimulus presentations. C) ERPs of experimental blocks averaged over electrode positions P7 and P8 contralateral to stimulus presentations. D) ERPs of experimental blocks at CPz.

The contralateral P1 peaked somewhat earlier (120 ms) than the ipsilateral P1 (135 ms; (Figures 5A and 5B, respectively). Stimuli containing large letters (i.e. large flanker trials or large target trials), elicited a more positive P1 than stimuli containing small letters (F(1, 15) = 5.29, p = .036 and F(1, 15) = 13.58, p = .002, for the contra- and ipsilateral P1, respectively), reflecting the direct effect of stimulus size. No significant interactions were observed.

#### N1

The contralateral N1 peaked approximately 160 ms after stimulus onset (Figure 5C). The amplitude of the N1 decreased with increasing time on task (F(4, 60) = 5.66, p = .001). Furthermore, we observed a smaller N1 peak for large compared to small stimuli (F(1, 15) = 12.51, p = .002), still indicative of the direct effect. Furthermore, a smaller N1 peak for incongruent than for congruent stimuli (F(1, 15) = 10.53, p = .005). No significant interactions were observed.

**Table 2:** Main and interaction effect on mean ERP amplitudes on CPz in the 300–400 ms interval and on the P3b.

		Reaction times		Accuracy	
Factors	df	F	p	F	p
TOT	4, 60	5.854	.006	2.449	
Cong	1, 15	6.136	.026	.005	
Size	1, 15	3.376		34.166	<.001
TOT×Cong	4, 60	.063		1.492	
TOT×Size	4, 60	1.957		2.054	
Cong×Size	1, 15	.002		4.085	
TOT×Cong×Size	4, 60	1.754		1.179	
Rel×Size	1, 15	4.949	.042	39.101	<.001
TOT×Rel×Size	4, 60	3.608	.011	2.111	
Cong×Rel×Size	1, 15	.442		1.810	
TOT×Cong×Rel×Size	4, 60	1.303		1.418	

TOT  =  time on task, Cong  =  congruency, Rel  =  relevance. Only significant p-values are listed.

**Figure 6:**
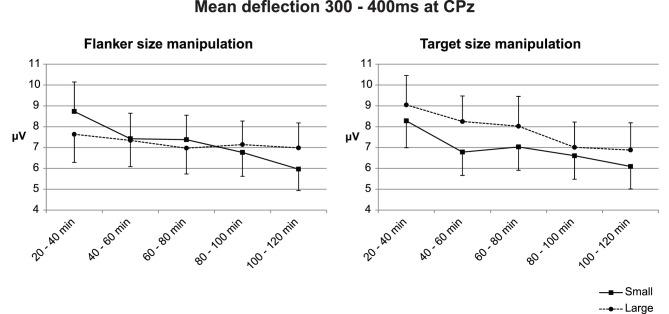
Interaction plots of the averaged voltage at the ascending flank of the P3b at CPz. These plots show a relatively constant difference in the ERP deflection for large versus small targets, while the ERP deflection for flankers changes from a smaller deflection for larger stimuli at the beginning to a larger deflection for the same stimuli at the end. Error bars represent standard errors.

#### P2

The P2 was most pronounced at PO3 and PO4, contralateral to stimulus presentation location, where it peaked around 230 ms after stimulus onset. The amplitude of the P2 was affected by stimulus congruency (F(1, 15) = 5.13, p = .039), resulting in a more positive peak for incongruent stimuli than for congruent stimuli. No main effects were observed for the factors time on task or size and no significant interactions were observed.

#### N2b

In the present experiment, the N2b peaked at Cz around 280 ms. No significant main effects or interaction effects were observed for the involved factors on this ERP.

#### P3b

The P3b peaked around 440 ms. The amplitude of the P3b was neither modulated by time on task (F(4, 60) = 2.45, n.s.), nor by congruency (F(1, 15) = .01, n.s.). However, there was an effect of stimulus size (F(1, 15) = 34.17, p<.001), reflected in a more positive deflection for large stimuli. This effect was more pronounced when the target size was manipulated than when the flanker size was manipulated (F(1, 15) = 39.10, p<.001), suggesting attention towards relevant information. The three-way interaction between relevance, size and congruency, which was observed in the behaviouralmeasurements, was not found on this ERP component (F(1, 15) = 1.81, n.s.). An overview of all observed effects on this component can be found in Table 2.

#### 300–400 ms interval

Visual inspection indicated that time on task had an effect on the ascending flank of the P3b, approximately between 300 and 400 ms at CPz (Figure 5D). Because Boksem et *al.* [6] found their N2b effects in approximately the same latency range, we decided to include mean amplitudes within this range in our analyses. To check whether modulation of attention on the ERP occurred in this interval, we investigated the three-way interaction between time on task, relevance and size. Statistical analysis revealed a significant interaction (F(4, 60) = 3.61, p = .011, see Figure 6). To seek confirmation that this effect was due to mental fatigue, we checked whether the effect was linear over time, which it was (F(1, 15) = 17.76, p = .001). On trials in which target size was manipulated, large targets generated a more positive deflection (F(1, 15) = 5.40, p = .035), however, this effect did not interact with time on task (F(4, 60) = .92, n.s.). On trials in which the size of the flankers was manipulated, there was an interaction between size and time on task where large flankers initially generated a more negative deflection, while they generated a more positive deflection than small flankers with increasing mental fatigue (F(4, 60) = 4.80, p = .002).

In addition, large targets generated a more positive deflection than small targets (F(1, 15) = 5.85, p = .029), while no size difference was observed for differences in flanker size (F(1, 15) <.01, n.s.). Furthermore, a main effect of time on task (F(4, 60) = 5.85, p = .006) was reflected in a decreased amplitude in this latency area from 8.42 µV (SD = 5.54) to 6.48 µV (SD = 4.62) and incongruent stimuli generated a more positive deflection (M = 7.68 µV, SD = 4.93) than congruent stimuli (M = 6.95 µV, SD = 4.78) (F(1, 15) = 6.14, p = .026). No main effect of stimulus size was observed. An overview of all observed effects in this interval can be found in Table 2.

## Discussion

In this study we investigated whether and how mental fatigue affects selective attention in visual processing by examining differences in processing of task relevant versus task irrelevant information. We studied how this influences event-related potentials, as well as behavioural measures. We used an adapted version of the Eriksen flanker task, which continued for two hours to induce mental fatigue. The observed decrease in accuracies and increase in reaction times and resistance to continue task performance, are in line with the typical time on task effects observed in studies of mental fatigue [40]. Therefore we conclude that the experimental paradigm succeeded in inducing mental fatigue. In addition to the observed effects of mental fatigue, the behavioural results showed congruency effects, typically observed in flanker tasks, which consist of slower and less accurate responses for incongruent than for congruent trials [32].

An important finding of this experiment was observed in the ERP of midline parieto-central locations, between 300 and 400 ms after stimulus onset. Here, an interaction between relevance – target versus flanker manipulation – and size was present. This effect indicated that at this stage the brain has made a selection of which visual information was relevant to the task and which was not, and is attempting to suppress the irrelevant signals, while relevant signals are being enhanced. With increasing mental fatigue, induced by continuous task performance, we observed changes in this interaction, suggesting changes in attentional modulations in stimulus processing. These changes were mainly related to the processing of irrelevant information; whereas for the relevant targets no changes were observed in brain activity with increasing time on task, for the irrelevant flankers the difference in brain activity elicited by large and small flankers changed gradually from negative to positive. This change might reflect a reduction of the suppression of irrelevant information, indicating that our information processing system seems to become less able to block out irrelevant information with increasing mental fatigue. However, the fatigued brain seems to have no problems processing the relevant information.

This interaction between time on task, relevance and size may at first glance seem to be driven solely by the absence of an effect of time on task in the large distracter condition. However, this is purely because a main effect of time on task coincides with the observed three-way interaction. Because reduced peak sizes with time on task were also present on the N1, this main effect of time on task may reflect an overall down-regulation of neural activity, perhaps due to decreased arousal, which would be in line with norepiniphrine models of mental fatigue (e.g. [2]). However, this main effect may also be due to a shift in response latencies, on which later more.

If indeed suppression of irrelevant information is hampered with increasing mental fatigue, one can expect that responses will increasingly be based on irrelevant information. According to so-called dual route models [41]–[42], this will have an effect on the amount of conflict at the response level. In the incongruent condition, less suppression of irrelevant information with increasing time on task will lead to increased response conflict. In the congruent condition, this effect may lead only to a slight facilitation. Besides by the ability to suppress irrelevant information, the amount of response conflict is also determined by the proportion of relevant versus irrelevant information arriving at the response stage and thus it will rely on the relative size of the target, compared to the flankers. This relative target size effect can be expected to be additive to the congruency effect, resulting in increased detrimental effects of incongruent stimuli with increasing time on task when the flankers are relatively large, compared to when they are relatively small.

What we observed in behaviour is that when relevant information was presented in a larger font than distracting information, people reacted faster and more accurately than if relevant information was presented in a smaller font. This effect was especially clear in incongruent trials. The size-related effects on accuracy levels observed in incongruent trials increased with time on task, while for congruent trials they remained similar (flanker size manipulation) or even decreased (target size manipulation) during the experimental session. This suggests, in line with the ERP findings, that with increasing mental fatigue, irrelevant information is interfering more strongly with response decision processes. The fact that these changes were only found for accuracy measures and not for reaction times, suggests that fatigue induced attentional modulations are primarily related to the suppression of irrelevant signals and prevention of errors.

Besides these interactions, a main effect of stimulus size was observed as early as 120 ms in the form of modulations of early ERP components (e.g., the contralateral and ipsilateral P1). These early components were not modulated by time on task. The earliest component that did show time on task effects was the N1. An interaction between size and stimulus relevance was not observed until 300 ms post-stimulus. We can thus conclude that, as expected, stimulus size indeed modulated early ERP components directly, while indirect size effects related to modulations of neural activation by attention become apparent later in time.

The fact that the selection of the 300 to 400 ms interval was based on visual inspection raises the concern for a selection bias. However, this selection was performed based on a main effect of time-on-task only. Therefore, it might be argued that the selection bias is only applicable to this main effect and not to the interaction on which we base our conclusions. Furthermore, there were theoretical grounds to choose the interval, because of the temporal overlap with Boksem et *al.*’s [6] N2b interval, which ran from 320 to 410 ms post-stimulus. Actual comparisons of our time on task effects on the ERPs brain activation with the results of Boksem et *al.* [6], showed similar effects of mental fatigue in both studies. In both studies attention related changes with time on task were observed within approximately the same interval and on comparable electrode positions. However, the peak latencies of identified ERP components in their study are different from ours. For example, they found a main effect of time on task on the N2b, which was not observed on the N2b peak in the current experiment, but did occur in the same time range. The question is whether the observed effects are indeed reflecting specific modulations of the N2b or whether they are coincidentally overlaying this ERP component in time.

An issue concerning the observed ERP effect in the 300 to 400 ms interval is that it is positioned on the rising flank of the P3b and therefore is sensitive to latency effects of the P3b peak. If the P3b peak latency increases, the steepness of the rising flank decreases, and hence the mean amplitude also decreases. P3 latency has since long been reported to be linked to response latencies (e.g. [43]–[44]), and Figure 5D does show an increase in peak latencies with increasing time on task, while response latencies also increase (Figure 2A). However, the ERP results show a three way interaction between time on task, relevance and size, which cannot be observed in the reaction times, thus the observed ERP effect, on which the conclusions are based, is not linked to response latencies and therefore more likely reflect an inherently different brain process. Furthermore, in the paradigm used in the present experiment, the target-manipulation-trials and flanker-manipulation-trials are qualitatively the same; relevant letters within the stimuli are either larger or smaller than irrelevant letters. Whether or not ERP effects in the 300 to 400 ms interval are caused by P300 peak latency effects, the interaction between relevance and size can only be explained as an attention effect, because there is clearly a selection of relevant versus irrelevant information. The observed ERP-effects must therefore be caused by modulation of attention.

To summarise: our experiment shows both the expected early effect of the size manipulation as well as the late effect, which indicated that a selection of relevant versus irrelevant information has been made. This late effect was influenced by mental fatigue, confirming that mental fatigue indeed causes changes selective attention and furthermore that this is not so much due to changes in the processing of relevant signals, but to changes in the suppression of irrelevant signals. Mentally fatigued individuals were shown to have problems blocking out distracting stimuli, leading to an increase in the number of errors. Changes in reaction times, however, seem to be caused by other, non-attention-related processes.

## References

[1] van der Linden D, Frese M, Meijman TF (2003) Mental fatigue and the control of cognitive processes: effects on perseveration and planning. Acta Psychol (Amst) 113: 45–65. 12679043 10.1016/s0001-6918(02)00150-6

[2] van der Linden D (2011) The urge to stop: the cognitive and biological nature of acute mental fatigue. In: Ackerman PL, editor. Cognitive fatigue: multidisciplinary perspectives on current research and future applications. Washington: American Psychological Association. 149–164.

[3] Meijman TF (1997) Mental fatigue and the efficiency of information processing in relation to work times. Int J Ind Ergon 20: 31–38.

[4] Bartlett FC (1943) Ferrier lecture: fatigue following highly skilled work. Proc R Soc Lond B Biol Sci 131: 247–257.

[5] van der Linden D, Eling P (2006) Mental fatigue disturbs local processing more than global processing. Psychol Res 70: 395–402. 15968553 10.1007/s00426-005-0228-7

[6] Boksem MAS, Meijman TF, Lorist MM (2005) Effects of mental fatigue on attention: an ERP study. Cognitive brain research 25: 106–117. 10.1016/j.cogbrainres.2005.04.011 15913965

[7] Eason RG (1981) Visual evoked potential correlates of early neural filtering during selective attention. Bull Psychon Soc 18: 203–206.

[8] Harter MR, Aine CJ (1984) Brain mechanisms of visual selective attention. In: Parasuramaran R, Davies D, editors. Varieties of attention. : Academic Press. 293–321.

[9] Hillyard SA, Mangun GR (1987) Commentary: sensory gating as a physiological mechanism for visual selective attention. In: Johnson R, Rohrbaugh J, Parasuramaran R, editors. Current trends in event-related potential research (eeg suppl. 40). : Elsevier. 138–145. 3480183

[10] Hillyard SA, Vogel EK, Luck SJ (1998) Sensory gain control (amplification) as a mechanism of selective attention: electro-physiological and neuroimaging evidence. Philos Trans R Soc Lond B Biol Sci 353: 1257–1270. 9770220 10.1098/rstb.1998.0281 PMC1692341

[11] Gazzaley A, Cooney JW, McEvoy K, Knight RT, D’Esposito M (2005) Top-down enhancement and suppression of the magnitude and speed of neural activity. J Cogn Neurosci 17: 507–517. 15814009 10.1162/0898929053279522

[12] Kim YJ, Grabowecky M, Paller KA, Muthu K, Suzuki S (2007) Attention induces synchronization-based response gain in steady-state visual evoked potentials. Nat Neurosci 10: 117–125. 17173045 10.1038/nn1821

[13] Ito M, Gilbert CD (1999) Attention modulates contextual influences in the primary visual cortex of alert monkeys. Neuron 22: 593–604. 10197538 10.1016/s0896-6273(00)80713-8

[14] McAdams CJ, Maunsel JH (1999) Effects of attention on orientation-tuning functions of single neurons in macaque cortical area v4. J Neurosci 19: 431–441. 9870971 10.1523/JNEUROSCI.19-01-00431.1999 PMC6782389

[15] Mehta AD, Ulbert I, Schroeder CE (2000) Intermodal selective attention in monkeys. Distribution and timing of effects across visual areas. Cereb Cortex 10: 343–358. 10769247 10.1093/cercor/10.4.343

[16] Motter BC (1993) Focal attention produces spatially selective processing in visual cortical areas v1, v2 and v4 in the presence of competing stimuli. J Neurophysiol 70: 909–919. 8229178 10.1152/jn.1993.70.3.909

[17] Roelfsema PR, Lamme VA, Spekreijse H (1998) Object-based attention in the primary visual cortex of the macaque monkey. Nature 395: 376–381. 9759726 10.1038/26475

[18] Vidyasagar TR (1998) Gating of neuronal responses in macaque primary visual cortex by an attentional spotlight. Neuroreport 9: 1947–1952. 9674572 10.1097/00001756-199806220-00006

[19] Brefczynski JA, DeYoe EA (1999) A physiological correlate of the ‘spotlight’ of visual attention. Nat Neurosci 2: 370–374. 10204545 10.1038/7280

[20] Martínez A, Anllo-Vento L, Sereno MI, Frank LR, Buxton RB, et al (1999) Involvement of striate and extrastriate visual cortical areas in spatial attention. Nat Neurosci 2: 364–369. 10204544 10.1038/7274

[21] Shulman GL, Corbetta M, Buckner RL, Raichle ME, Fiez JA, et al (1997) Top-down modulation of early sensory cortex. Cereb Cortex 7: 193–206. 9143441 10.1093/cercor/7.3.193

[22] Somers DC, Dale AM, Seiffert AE, Tootell RBH (1999) Functional mri reveals spatially specific attentional modulation in human primary visual cortex. Proc Natl Acad Sci U S A 96: 1663–1668. 9990081 10.1073/pnas.96.4.1663 PMC15552

[23] Tootell RBH, Hadjikhani N, Hall EK, Marrett S, Vanduffel W, et al (1998) The retinotopy of visual spatial attention. Neuron 21: 1409–1422. 9883733 10.1016/s0896-6273(00)80659-5

[24] Di Russo F, Martínez A, Hillyard SA (2003) Source analysis of event-related cortical activity during visuo-spatial attention. Cereb Cortex 13: 486–499. 12679295 10.1093/cercor/13.5.486

[25] Martínez A, Di Russo F, Anllo-Vento L, Sereno MI, Buxton RB, et al (2001) Putting spatial attention on the map: timing and localization of stimulus selection processes in striate and extrastriate visual areas. Vision Res 41: 1437–1457. 11322985 10.1016/s0042-6989(00)00267-4

[26] Jehee JFM, Lamme VAF, Roelfsema PR (2007) Boundary assignment in a recurrent network architecture. Vision Res 47: 1153–1165. 17368500 10.1016/j.visres.2006.12.018

[27] Lamme VAF (1995) The neurophysiology of figure-ground segregation in primary visual cortex. J Neurosci 15: 1605–1615. 7869121 10.1523/JNEUROSCI.15-02-01605.1995 PMC6577835

[28] Lamme VAF, Rodriguez-Rodriguez V, Spekreijse H (1999) Separate processing dynamics for texture elements, boundaries and surfaces in primary visual cortex of the macaque monkey. Cereb Cortex 9: 406–413. 10426419 10.1093/cercor/9.4.406

[29] Roelfsema PR, Lamme VAF, Spekreijse H, Bosch H (2002) Figure-ground segregation in a recurrent network architecture. J Cogn Neurosci 14: 525–537. 12126495 10.1162/08989290260045756

[30] Smith AT, Singh KD, Williams AL, Greenlee MW (2001) Estimating Receptive Field Size from fMRI Data in Human Striate and Extrastriate Visual Cortex. Cereb Cortex 11: 1182–1190. 11709489 10.1093/cercor/11.12.1182

[31] Scholte HS, Jolij J, Fahernfort JJ, Lamme VAF (2008) Feedforward and recurrent processing in scene segmentation: electroencephalography and functional magnetic resonance imaging. J Cogn Neurosci 20: 2097–2109. 18416684 10.1162/jocn.2008.20142

[32] Eriksen BA, Eriksen CW (1974) Effects of noise letters upon the identification of a target letter in a nosearch task. Percept Psychophys 16: 143–149.

[33] Busch NA, Debener S, Kranczioch C, Engel AK, Herrmann CS (2004) Size matters: effects of stimulus size, duration and eccentricity on the visual gamma-band response. Clin Neurophysiol 115: 1810–1820. 15261860 10.1016/j.clinph.2004.03.015

[34] Roelfsema PR, Lamme VAF, Spekreijse H, Bosch H (2002) Figure-ground segregation in a recurrent network architecture. J Cogn Neurosci 14: 525–537. 12126495 10.1162/08989290260045756

[35] Ratcliff R, McKoon G (2008) The Diffusion Decision Model: Theory and Data for Two-Choice Decision Tasks. Neural Comput 20: 873–922. 18085991 10.1162/neco.2008.12-06-420 PMC2474742

[36] Aine CJ, Supek S, George JS, Ranken D, Lewine J, et al (1996) Retinotopic organization of human visual cortex: departures from the classical model. Cereb Cortex 6: 354–361. 8670663 10.1093/cercor/6.3.354

[37] Clark VP, Fan S, Hillyard SA (1995) Identification of early visual evoked potential generators by retinotopic and topographic analyses. Hum Brain Mapp 2: 170–187.

[38] Zijlstra FRH, Van Doorn L (1985) The construction of a subjective effort scale.

[39] Gratton G, Coles MGH, Donchin E (1983) A new method for off-line removal of ocular artifact. Electroencephalogr Clin Neurophysiol 55: 468–484. 6187540 10.1016/0013-4694(83)90135-9

[40] Lorist MM, Faber LG (2011) Consideration of the influence of mental fatigue on controlled and automatic cognitive processes and related neuromodulatory effects. In: Phillip L Ackerman, editor. Cognitive fatigue: multidisciplinary perspectives on current research and future applications. Washington: American Psychological Association. 105–126.

[41] Kornblum S, Hasbroucq T, Osman A (1990) Dimensional overlap: Cognitive basis for stimulus-response compatibility- A model and taxonomy. Psychol Rev 97: 253–270. 2186425 10.1037/0033-295x.97.2.253

[42] Ridderinkhof KR (1997) Commentary on Lu: A dual-route processing architecture for stimulus-response correspondence effects. In: Hommel B, Prinz W, editors. Theoretical issues in stimulus-response compatibility. Amsterdam: Elsevier Science. 119–131.

[43] Verleger R, Jaśkowski P, Wascher E (2005) Evidence for an integrative role of P3b in linking reaction to perception. J Psychophysiol 19: 165–181.

[44] McCarthy G, Donchin E (1981) A metric for thought: A comparison of P300 latency and reaction time. Science 211: 77–80. 7444452 10.1126/science.7444452

